# Supplementary material for: A quick guide for student-driven community genome annotation
Source: PLoS Comput Biol. 2019 Apr 3;15(4):e1006682. doi: 10.1371/journal.pcbi.1006682 (PMC6447164; doi:10.1371/journal.pcbi.1006682)
Supplement: S1 Table — (DOCX) [file pcbi.1006682.s001.docx]

Supplementary Table 1: List of suggested personnel, hardware and software resources for community curation projects of differing scope. Laptops or desktops for individual annotators are required for all project types. This does not included resources needed for running gene prediction pipelines. ^1^ Artemis is a stand-alone tool so we recommend its use for only small scale projects. Larger projects should use Apollo so that all the data is stored in a central location.

| **Project scope** | **Software** | **Hardware** | **Infrastructure** |
| --- | --- | --- | --- |
| Single institution  (option 1) | Apollo server  (<http://genomearchitect.org/>)  and any web browser | Desktop web server for hosting Apollo installation on intranet. | Instructor and teaching assistants for managing lab sessions. IT support might be needed for installation and system administration. |
| Single institution  (option 2) | Artemis^1^ ([https://www.sanger.ac.uk/ science/tools/artemis](https://www.sanger.ac.uk/%20science/tools/artemis)) | NA | Instructor and teaching assistants for managing lab sessions. |
| Single institution  (option 3) | Depends on project | NA | Groups can join public annotation projects with web-based platforms such as i5k (<https://i5k.nal.usda.gov/>) or Genomics Education Partnership (<http://gep.wustl.edu/>). Instructor and teaching assistants for managing local lab sessions. |
| Single institution  (option 4) | G-OnRamp server (<http://gonramp.wustl.edu/>) | Web server for hosting G-OnRamp installation on intranet. | Instructor and teaching assistants for managing lab sessions. IT support might be needed for installation and system administration. |
| Multiple institutions  (option 1) | Apollo server (<http://genomearchitect.org/>) and any web browser | Web server for hosting Apollo installation on internet. | Instructor and teaching assistants for managing local lab sessions. Regular video conferences for entire team. IT support might be needed for installation and system administration. |
| Multiple institutions  (option 2) | Depends on project | NA | Groups can join public annotation projects with web-based platforms such as i5k (<https://i5k.nal.usda.gov/>) or Genomics Education Partnership (<http://gep.wustl.edu/>). Instructor and teaching assistants for managing local lab sessions. Regular video conferences for entire team. |
| Multiple institutions  (option 3) | G-OnRamp server (<http://gonramp.wustl.edu/>) | Web server for hosting G-OnRamp installation on internet. | Instructor and teaching assistants for managing lab sessions. IT support might be needed for installation and system administration. |
