# Supplementary material for: A quick guide for student-driven community genome annotation
Source: PLoS Comput Biol. 2019 Apr 3;15(4):e1006682. doi: 10.1371/journal.pcbi.1006682 (PMC6447164; doi:10.1371/journal.pcbi.1006682)
Supplement: S2 Table — (DOCX) [file pcbi.1006682.s002.docx]

Supplementary Table 2: Websites with training resources and guidelines for genome annotation

| **Source** | **Description** | **Link** |
| --- | --- | --- |
| PlantGDB | Annotation tutorials | <http://www.plantgdb.org/tutorial/annotatemodule/studentsection.html> |
| Genomics Education partnership | Tutorials about basics including NCBI blast and identifying homology | <http://gep.wustl.edu/curriculum/course_materials_WU/annotation/tutorials_and_walkthroughs> |
| i5k | Overview of manual curation procedures for insect genomes | <https://i5k.nal.usda.gov/manual-curation-overview> |
| Apollo | User guide | <http://genomearchitect.github.io/users-guide/> |
| Okinawa Institute of Science and Technology Graduate University | Introduction to genetics, basic techniques and evolutionary analysis | <http://ecoevo.unit.oist.jp/lab/?page_id=50> |
| FlyBase | Gene Model Annotation Guidelines | <https://wiki.flybase.org/wiki/FlyBase:Gene_Model_Annotation_Guidelines> |
| *Diaphorina citri* annotation project | Annotation guidelines, tutorial and exercises in supplementary data | <https://citrusgreening.org/annotation/updates> and Saha et al., 2017 [9] |
