# Supplementary material for: A quick guide for student-driven community genome annotation
Source: PLoS Comput Biol. 2019 Apr 3;15(4):e1006682. doi: 10.1371/journal.pcbi.1006682 (PMC6447164; doi:10.1371/journal.pcbi.1006682)
Supplement: S1 File — (DOCX) [file pcbi.1006682.s003.docx]

**Supplementary File 1**

**Concept inventory test for genome annotation to be taken by students at the end of the course. The questions are organized according to the proposed annotation workflow (Figure 1).**

**Name:**

**Previous classes (biology and bioinformatics):**

**____________________**

**Background**

**Questions**

**Circle correct answer. Please answer essay type questions in 50-100 words**

**1. Amino acids are linked together to form proteins by specific bonds called:**

A) Peptide bonds. B) Nitrogen bonds. C) Hydrogen bonds. D) Hydrogen & Nitrogen bonds

**2. The enzyme which builds mRNA strand complimentary to DNA transcription unit is called:**

A) DNA polymerase. B) RNA polymerase. C) Helicase. D) DNA ligase.

**3. The part of gene that codes for a protein is:**

A) Exon. B) Intron. C) Regulatory sequence. D) None of these

**4. With regard to the DNA structures:**

A) A and G are Pyrimidine bases. B) A and T are purine bases.

C) C and G are Pyrimidine bases. D) A and G are purine bases

**5. What are the repeating units of DNA?**

A) phosphate molecules. B) nucleotides. C) bases. D) sugar molecules.

**6. Chromosomes are more clearly visible during**

A) Interphase. B) Prophase. C) Metaphase. D) Anaphase

**7. The haploid (n) chromosome number in normal Human is:**

A) 16. B) 23. C) 50. D) 36.

**8. The triplet code of CAT in DNA is represented as ______ in mRNA.**

A) GAA B) CAT C) GUA D) GTA

**10. In DNA the amount of adenine (A) is ____________ the amount of thymine (T).**

A) much greater than. B) much less than. C) about the same as. D) shows no relationship to.

**11. What are some major difficulties associated with genome sequencing and assembly?**

**12. Provide a description of RNA-seq analyses and why would you use this method. How are RNA-seq reads critical to gene prediction and correction?**

**Finding orthologs**

**Questions**

**Circle correct answer. Please answer essay type questions in 50-100 words**

**1. Please list 3 bioinformatics tools and databases used to analyze domains.**

**2. Usually E values smaller than a certain threshold are considered to demonstrate homology. This threshold is usually about**

A) about 10^4^ B) about 10^-4^ C) about 10^-40^

**3. If you want to BLAST the non-redundant database using a new protein sequence as query, which is the BEST search program to use?**

A) blastp B) blastn C) tblastx D) blastx E) PRSS F) blastq

**4. Some students still have difficulties to discriminate between the term homology (=shared ancestry) and significant similarity. Which of the following statements is correct:**

A) All complex sequences that show significant similarity in a pairwise sequence

comparison are homologous.

B) All homologous sequences show significant similarity in a pairwise sequence

comparison.

C) Both of the above statements are correct

**5. If you do a databank search using FASTA with an amino acid sequence as query and the only reported match has an E-value of 12, what does this mean for the homology of the two sequences?**

A) This proves (beyond reasonable doubt) that the two sequences are homologs.

B) the target sequence is a candidate for a homologous sequence, but an E-value of this magnitude does not prove homology.

C) this proves (beyond reasonable doubt) that the target sequence is not homologous to

the query.

D) None of the above.

**6. For the following printout from BLASTX (translated query against the protein database), explain each of the highlighted components (5 total**). **Write no more than 1 sentence for each one.**

**1**

**2**

gi|1778588|gb|AAB40867.1| Gene info homeodomain protein HoxA9 [Homo sapiens]

Length = 271

Score = 92.0 bits (227), Expect = 2e-17

Identities = 47/90 (52%), Positives = 54/90 (60%)

Frame = +2

Query: 553 PSENYXXXXXXXXXXXXXXXPCTPNPGLHEWTGQVSVRKKRKPYSKFQTLELEKEFLFNA 732

PSE P PN W S RKKR PY+K QTLELEKEFLFN

Sbjct: 169 PSEGAFSENNAENESGGDKPPIDPNNPAANWLHARSTRKKRCPYTKHQTLELEKEFLFNM 228

**3**

Query: 733 YVSKQKRWELARNLQLTERQVKIWFQNRRM 822

**5**

Y+++ +R+E+AR LTERQVKIWFQNRRM

Sbjct: 229 YLTRDRRYEVARLFNLTERQVKIWFQNRRM 258

**4**

**Apollo and manual curation**

**Questions**

**Circle correct answer. Please answer essay type questions in 50-100 words**

**1. Why is it helpful to have a set of *de novo* assembled (genome-independent) transcripts when annotating a genome?**

**2. How would you determine whether two predicted transcripts with different gene structure are actually different isoforms?**

**3. Which of the following best represents a consensus splice site (from 5’ to 3’)?**

**A) exon AT…………….………..GG exon**

**B) exon GT……………………….AG exon**

**C) intron AT……………………..GG intron**

**D) intron GT………………………AG intron**

**4. You are annotating gene X in from the unicorn genome. Your research indicates that orthologs of gene X have two conserved domains called ABC and DEF. Your analysis of the protein encoded by the computationally-predicted protein gene X in unicorns shows that domain ABC is present but domain DEF is not. What would you do next?**

**5. Please list three reasons why a gene model might be incorrectly truncated by a stop codon.**

**6. RNA-seq reads commonly extend far past the start and stop codons of genes. What is the role of this extended mRNA sequence? Would you expect all mRNA sequences from the same gene to be identical? Please explain and include a diagram.**

**6. What is the major difference between a BLAST and a BLAT search?**

**7. What is a single nucleotide polymorphism? Why is the identification of these critical to bioinformatics and to biology?**

**Reporting**

**Questions**

**Please answer essay type questions in 80-100 words**

**1. How can you use a phylogenetic tree to resolve the evolutionary history of a gene family? How can you use orthologous sequences for this analysis?**

**2. What is an Official gene set? How is it useful for data distribution, sharing and versioning?**
